# *Ascophyllum nodosum* Seaweed Extract Alleviates Drought Stress in *Arabidopsis* by Affecting Photosynthetic Performance and Related Gene Expression

**DOI:** 10.3389/fpls.2017.01362

**Published:** 2017-08-03

**Authors:** Antonietta Santaniello, Andrea Scartazza, Francesco Gresta, Elena Loreti, Alessandro Biasone, Donatella Di Tommaso, Alberto Piaggesi, Pierdomenico Perata

**Affiliations:** ^1^Plant Lab, Institute of Life Sciences, Scuola Superiore Sant’Anna Pisa, Italy; ^2^Institute of Agroenvironmental and Forest Biology, Consiglio Nazionale delle Ricerche Rome, Italy; ^3^Institute of Agricultural Biology and Biotechnology, Consiglio Nazionale delle Ricerche Pisa, Italy; ^4^Global R&D, Valagro SpA Atessa, Italy

**Keywords:** antioxidants, drought tolerance, gas exchanges, mesophyll conductance, non-photochemical quenching, photochemical efficiency, stomatal conductance, water use efficiency

## Abstract

Drought represents one of the most relevant abiotic stress affecting growth and yield of crop plants. In order to improve the agricultural productivity within the limited water and land resources, it is mandatory to increase crop yields in presence of unfavorable environmental stresses. The use of biostimulants, often containing seaweed extracts, represents one of the options for farmers willing to alleviate abiotic stress consequences on crops. In this work, we investigated the responses of *Arabidopsis* plants treated with an extract from the brown alga *Ascophyllum nodosum* (ANE), under drought stress conditions, demonstrating that ANE positively influences *Arabidopsis* survival. Pre-treatment with ANE induced a partial stomatal closure, associated with changes in the expression levels of genes involved in ABA-responsive and antioxidant system pathways. The pre-activation of these pathways results in a stronger ability of ANE-treated plants to maintain a better photosynthetic performance compared to untreated plants throughout the dehydration period, combined with a higher capacity to dissipate the excess of energy as heat in the reaction centers of photosystem II. Our results suggest that drought stressed plants treated with ANE are able to maintain a strong stomatal control and relatively higher values of both water use efficiency (WUE) and mesophyll conductance during the last phase of dehydration. Simultaneously, the activation of a pre-induced antioxidant defense system, in combination with a more efficient energy dissipation mechanism, prevents irreversible damages to the photosynthetic apparatus. In conclusion, pre-treatment with ANE is effective to acclimate plants to the incoming stress, promoting an increased WUE and dehydration tolerance.

## Introduction

The consumption of water by human is mainly driven by the activities aimed at food production, since irrigated agriculture is extremely important for global food provision. Global climate changes predict depletion in water resources availability (Turner and Meyer, 2011), making the improvement of plant water use efficiency (WUE) mandatory (Araus, 2004; Katerji et al., 2008; Morison et al., 2008; Geerts and Raes, 2009). Drought is defined as one of the most limiting factors for plant growth and yield, which causes changes at molecular and physiological level. These changes vary from morphology to the expression of genes and include stomatal response, metabolic adjustments and changes in photosynthesis rate (Chaves, 1991; Chaves et al., 2009). Most of these processes are regulated by the activity of a drought-induced hormone, abscisic acid (ABA) (Assmann, 2003; Jakab et al., 2005; Cutler et al., 2010; Lee and Luan, 2012), which plays a key role in water stress responses (Shinozaki et al., 2003; Bauer et al., 2013; Ng et al., 2014) and tolerance (Nakashima et al., 2014). Indeed, during water stress, the increased concentration of ABA triggers the transcriptional activation of several genes, which are involved in downstream responsive pathways. According to their supposed functions, these genes are classified into two groups. The ABA-responsive genes belong to the first group, which codify for structural proteins, downstream players in the stress response pathway, such as osmoregulatory genes, aquaporins, late embryogenesis abundant (LEA) and antioxidant proteins (Bailly et al., 2001; Breton et al., 2003; Shinozaki and Yamaguchi-Shinozaki, 2007). The second group counts early response transcriptional activators, transcription factors (*bZIP, WRKY, MYB, AP2/EREBP*) and protein kinases, which are involved in important regulative pathways of drought tolerance (Abe et al., 1997; Marè et al., 2004; Song et al., 2005). Drought stress influences plant primary processes such as photosynthesis and cell growth (Chaves, 1991; Chaves et al., 2009), through direct and/or secondary effects. Primary effects are the reduction of stomatal (g_s_) (Osakabe et al., 2014) and mesophyll (g_m_) conductance to carbonic dioxide (Flexas et al., 2016), which determines a lower availability of CO_2_ at the carboxylation site in the chloroplast (Sorrentino et al., 2016). Moreover, reduced CO_2_ assimilation increases the excess of excitation energy at photosystem II (PSII), which results in a strong alteration of photosynthetic metabolism (Lawlor and Cornic, 2002), with consequences on ribulose 1,5-bisphosphate carboxylase/oxygenase (Rubisco) protein quantity (Bota et al., 2004) and activity (Parry et al., 2002). Secondary effects are related to the consequent overproduction of reactive oxygen species (ROS), such as hydrogen peroxide, hydroxyl and superoxide anion radicals (Apel and Hirt, 2004; Noctor et al., 2014). High levels of ROS in the cell result in extensive damage to DNA, proteins and lipids, causing a metabolic dysfunction which leads to plant death (Gill and Tuteja, 2010; Anjum, 2015). To prevent these permanent damages to the photosynthetic apparatus, plants evolved efficient and complex protective mechanisms, for triggering adaptive responses. Plants have developed enzymatic and non-enzymatic antioxidative systems, involved in a fine tuning of ROS. Indeed, low ROS levels act as signal components in the regulation of important plant processes, included stress pathways (Baxter et al., 2014; Das and Roychoudhury, 2014). ROS-scavenging enzymes of plants include catalase, superoxide dismutase, glutathione-*S*-transferase, guaiacol peroxidase, and ascorbate peroxidase (You and Chan, 2015). Non-enzymatic antioxidant mechanisms involve low molecular weight compounds, such as ascorbic acid, reduced glutathione, flavonoids, and carotenoids (Foyer and Noctor, 2005; Gill and Tuteja, 2010).

When absorbed light energy exceeds the amount used by photosynthesis (Ruban et al., 2012), thermal dissipation of the excitation energy occurs, through the so-called non-photochemical quenching (NPQ) of chlorophyll a (Chl a) fluorescence, which protect the photosynthetic apparatus from damages due to the over-excitation of PSII reaction centers. These processes involve the xanthophyll cycle, the PSII-related protein PsbS, the water–water cycle and the dissociation of the photosynthetic reaction centers from light-harvesting complexes (Funk et al., 1995; Niyogi et al., 2004; Demmig-Adams and Adams, 2006; Roach and Krieger-Liszkay, 2012).

A major challenge for agriculture is how to improve the present food for feeding an increasing world’s human population, while crop yield per area is decreasing due to land desertification (Hatfield et al., 2011; Flexas et al., 2016). In order to improve WUE, two major approaches can be used: (i) traditional breeding and/or biotechnological approach (Medrano et al., 2009; Flexas et al., 2010) and (ii) development of agronomic practices (Tilman et al., 2002; Blum, 2009; Tallec et al., 2013), such as the use of specific products aimed to increase drought tolerance. In recent years, the use of biostimulants, often based on natural extracts, such as from seaweeds, has been proposed as a sustainable strategy for improving crop yields without adversely impacting on the environment (Jayaraj et al., 2008; Khan et al., 2009; Hernández-Herrera et al., 2014).

The use of *Ascophyllum nodosum* extracts in agriculture is largely documented, as an organic and mainstream biostimulant for many varieties of crop species (MacKinnon et al., 2010; Craigie, 2011; Spann and Little, 2011; Povero et al., 2016), also for its properties to increase stress tolerance in treated plants (Zhang and Ervin, 2004; Spann and Little, 2011; Petrozza et al., 2014). These effects are due to a complex assortment of bio-stimulatory molecules, which includes micronutrients, vitamins and complex organic compounds (Craigie, 2011). Notwithstanding the increasing number of papers related to this topic (Rayirath et al., 2009; Jithesh et al., 2012; Nair et al., 2012), the mode of action of the most part of the chemical components of these extracts remains not well-characterized yet.

The main aim of the present study was to investigate the effects of an *Ascophyllum nodosum* extract (ANE) on the regulation of water stress responses in *Arabidopsis* plants, in terms of both photosynthesis performance and impact on gene expression. So far, many works have analyzed the effect of ANE treatments on crop performance under abiotic stress conditions, mainly focusing on gas exchanges or gene expression measurements separately (Rayirath et al., 2009; Spann and Little, 2011; Jithesh et al., 2012; Nair et al., 2012; Xu and Leskovar, 2015; Goñi et al., 2016). However, to the best of our knowledge, this is the first time that phenotypic responses, stomatal and non-stomatal factors, specific antioxidant and photosynthetic-related genes were simultaneously investigated, in order to obtain an integrated view of the treatment effects on drought-stress tolerance from the genes to the plant.

## Materials and Methods

### Plant Material and Growing Conditions

Experiments were carried out using *Arabidopsis thaliana* accession Columbia-0 (Col-0). Plants were grown in polystyrene trays, containing 30 rock-wool plugs (Grodan, FL, United States) in hydroponic system (Gibeaut et al., 1997), changing the solution weekly. For all the experiments, the trays were kept in growth chamber with a photoperiod of 12-h light/12-h dark (100 μmol photons m^-2^ s^-1^), 23/18°C day/night of temperature and 50 ± 5% relative humidity (RH).

### Treatments and Stress Conditions

After sowing, trays were incubated for 2 days at 4°C for vernalization and then transferred to the growth chamber. Soluble extract powder obtained from *Ascophyllum nodosum* (provided by Algea, Valagro S.p.A) was used for the treatment. After 20 days of growth, a treatment with *Ascophyllum nodosum* extract (ANE) was performed, dissolving in the hydroponic medium 3 g L^-1^ of ANE and leaving the trays with plants for 5 days in this enriched solution, while the untreated plants were maintained in an unaltered hydroponic solution. Three blocks of thirty plants each were used for untreated and treated thesis, respectively. Water stress was induced by removing the trays from hydroponic solution and keeping them on a filter paper under growth chamber condition for 4 days. To minimize the influence of any micro-environmental gradients on drought stress responses, the relative position of the trays in the growth chamber was changed daily. To monitor the phenotypical effects induced by the stress on treated and untreated plants, during the 4 days of dehydration, pictures were taken daily and, simultaneously, the plot water content (PWC), leaf relative water content (RWC) and the number of dead plants were recorded. PWC was determined as PWC = W_plot_ - (W_poly_ + DW_wicks_), where W_plot_ was the weight of polystyrene plot with *Arabidopsis* plants, W_poly_ the weight of the polystyrene plot and DW_wicks_ the dry weights of the rock-wool wicks. To determine RWC five leaves (of the same age) from five plants in different positions on the tray and harvested at the same time of the day (10 a.m) were chosen. RWC (%) was calculated as [(FW - DW)/(TW - DW)] × 100, where FW is the fresh weight (FW), DW the dry weight and TW the turgid fresh weight. DW was determined by drying leaves for 72 h at 60°C, while for TW determination leaves were kept in Petri dish with distilled water for 24 h at 4°C.

### Chemical Composition Analysis of ANE

The *Ascophyllum nodosum* extract was manufactured using a proprietary process at acidic pH. Analyses of monosaccharides, oligosaccharides and amino acids were performed on an Agilent 1290 UHPLC system (Agilent, Waldbronn, Germany), coupled to triple Tof 5600+ mass spectrometry (ABSciex, Framingham, MA, United States).

The separation of 1-Phenyl-3-methyl-5-pyrazolone (PMP) labeled carbohydrates was carried out on a Waters Cortecs C18 2,1 × 150 2,7 μm (Waters Chromatography Div., Milford, MA, United States) with a flow rate of 0.25 mL min^-1^. Formic acid 5 mM ammonium formate 15 mM (solvent A), methanol 90% Formic acid 5 mM ammonium formate 15 mM (solvent B) were applied as mobile phases. The gradient was started after 1 min at 95% A, 1–30 min 95–40% A, 30–32 min 40–10% A and the column was equilibrated with 95% A for 20 min. The source conditions were as follow: temperature 500°C, GS1 50, GS 2 30, DP 60. The determination of labeled amino acids was carried out on a Waters Cortecs C18 2,1 × 150 2,6 μm (Waters Chromatography Div., Milford, MA, United States) with a flow rate of 0.25 mL min^-1^. ACN 5% ammonium formate 20 mM (solvent A), ACN 70% ammonium formate 20 mM (solvent B) were applied as mobile phases. The gradient was started after 2 min at 97% A, 2–20 min 97–50% A, 20–21 min 50–20% A and the column was equilibrated with 97% A for 20 min.

The quantitative analysis of monosaccharides were achieved in Tandem mass spectrometry (MS/MS) mode monitoring the loss of ethanol from the derivatives. The data were acquired using Analyst TF 1.7 (ABsciex, Framingham, MA, United States) and quantitative analysis was performed by Multi Quant 2.1 (ABSciex, Framingham, MA, United States). The hydrolysis of the sample to determine the total amino acids content was carried out by ETHOS One Microwave system [Milestone, Sorisole (BG), Italy].

Quantification of total carbohydrates after hydrolysis were performed starting from 300 mg of the prototype dissolved in 25 mL of Type III water. An aliquot of 250 μL was placed in a vial, diluted with 600 μL water and 150 μL of trifluoroacetic acid, sealed and let it stirred for 3 h at 120°C. The final solution was diluted to 5 mL with water and an aliquot of 50 μL was dried under nitrogen stream. The residue was dissolved by adding 100 μL ammonium hydroxide 1.5 M, 700 μL water, 100 μL of internal standard (galacturonic acid) and 500 μL of the methanolic solution of PMP (0,5 M). The solution was heated for 2 h at 75°C under magnetic stirring, cooled down and the excess of methanol was removed under nitrogen stream. The dried solution was brought at 1 mL with water. The excess of PMP was removed with methylene chloride (3 × 0,8 mL). The aqueous phase was diluted up to 5 mL with mobile phase, filtered through RC 0,2 μm membrane filter and analyzed by high-performance liquid chromatography coupled to electrospray ionization and quadrupole time-of-flight mass spectrometry (HPLC-ESI-QTOF, Agilent, Waldbronn, Germany). The concentration of the injected standard solutions range from 0,1 to 100 ppb.

To evaluate the presence of fucoidan and alginate, total and free carbohydrates were analyzed. The direct derivatization of the extract was carried out by dissolving the prototype in water following by direct labeling with PMP as reported above. Despite the presence of guluronic acid and mannuronic acid, their quantitative determination is not allowed because the lack of their commercial standards.

The free and total amino acids were labeled with DEEM according to Gómez-Alonso et al. (2007, pp 608–613) and quantified by mass spectrometry. The total amino acids content was determined after Microwave-assisted hydrolysis. Briefly 300 mg of sample was placed at the bottom of PTFE vessel, 100 mg of phenol, 10 mL Hydrochloric acid 6 M were added and before the sealing the atmosphere was saturated with gaseous nitrogen. The hydrolysis is carried out at 150°C for 3 h.

### RNA Isolation, cDNA Synthesis, and Real-Time qPCR Analysis

The RNA extraction was performed according to Paparelli et al. (2012). To check for RNA integrity, electrophoresis was performed for all RNA samples, using a 1% agarose gel followed by spectrophotometric quantification. TURBO DNA-free kit (Ambion^1^) was used to remove contaminant DNA. RNA was then reverse-transcribed using an iScript TM cDNA synthesis kit (BioRad Laboratories^2^). Expression analysis of *NCED3*, nine-*cis*-epoxycarotenoid dioxygenase 3 (*At3g14440*); *MYB60*, MYB domain protein 60 (*At1g08810*); *RAB18*, responsive to ABA18 (*At5g66400*); *RD29A*, responsive to desiccation 29A (*At5g52310*); *RBCS1A*, ribulose 1,5-bisphosphate carboxylase/oxygenase small subunit A (*At1g67090*); *RCA*, Rubisco activase (*At2g39730*); *PIP1;2*, plasma membrane intrinsic protein 1;2 (*At2g45960*); *βCA1*, β carbonic anhydrase 1 (*At3g01500*); *PsbS*, photosystem II subunit S (At1g44575); *VDE*, violaxanthin de-epoxidase (*At1g08550*); *DFR*, dihydro flavonol reductase (*At5g42800*); *SOD*, super oxide dismutase (*At1g8830*); *APX2*, ascorbate peroxidase 2 (*At3g09640*) and *ZAT10* (*At1g27730*), were performed by real-time PCR, using an ABI Prism 7300 sequence detection system (Applied Biosystems). For quantitative PCR 30 ng cDNA were processed with iQTM SYBR^®^ Green Supermix (Biorad Laboratories, Hercules, CA, United States). Expression of *UBQ10*-Ubiquitin10 (*At4g05320*), *TIP41*-*like* (*At4g34270*) and Unknown Protein (*At4g33380*) were used as endogenous controls. Relative expression levels were calculated using GeNorm^3^. To design specific gene primers, the QuantPrime Tool was used (^4^Arvidsson et al., 2008) and reported in **Table 1**. Gene expression analysis were performed during treatment time, starting from 1 to 5 days of treatment (1–5 DOT) and dehydration period, from 0 up to 4 days after dehydration (0–4 DAD). Time 0 is the drought stress starting time point and it corresponds exactly with the last day of *Ascophyllum nodosum* extract treatment. The samples were harvested cutting the entire rosette, immediately frozen in liquid N_2_, and stored at -80°C. Each rosette was processed as an independent replicate, using four biological replicates for each time point. Expression data, related to the treatment time (1–5 DOT, inset), were graphically shown in the figures only when, comparing untreated and ANE-treated samples, at least one time point was significantly different.

**Table 1:** List of specific primers forward (FW) and revers (RV) used for gene expression analysis.

Gene symbol	FW	RV
*NCED3*	TGAAGTCGTCGTGATAGGGTCCTG	ATCGGACGGCGAGTTGATTCAC
*RAB18*	TCTAGCTCGGAGGATGATGGAC	ACCGTAGCCACCACCAGCATCATATC
*RD29A*	TGGACAAAGCAATGAGCATGAGC	AGGTTTACCTGTTACGCCTGGTG
*RBCS1A*	TTCCTGACCTTACCGATTCC	GCATTGGGGTACTCCTTCTT
*RCA*	AGACCGTATCGGTGTCTGCAAG	CCCTCAAAGCACCGAAGAAATCG
*PIP1;2*	TGCCACTGATGCCAAGAGAAACG	AGGGAGCGGTGCTAGAATAGGAAC
*βCA1*	TGGACTTTCAGCCAGGAGATGC	CAACGCCACCGTATTTGACCTTG
*PsbS*	CATTGGAGCTCTCGGAGACAGAGGAA	CTCGTTCGCCTTCGTGAACCCAAACAAT
*VDE*	ATGACTGGTATATCCTGTCATC	CGTTCTAATGAATGTGCTGAAG
*DFR*	AGGAAGGAAGCTACGATGATGCC	TGTCGGCTTTATCACTTCGTTCTC
*SOD*	AACGGTTGCATGTCTACTGGTC	GTGATTGTGAAGGTGGCAGTTCC
*APX2*	CACAAGGAGCGTTCAGGATT	TGAGGGAACAAGAATCAAGGA
*ZAT10*	TCTCCGATTCCTCCTTTGTTCG	AGATCGCTTACCCTTTGTCCAG
*UBQ10*	GGCCTTGTATAATCCCTGATGAATAAG	AAAGAGATAACAGGAACGGAAACATAGT
*TIP41*-*like*	AGTGAGAGTCATGCCAAGCT	CAGTTGGTGCCTCATCTTCG
*Unknown protein*	AGTGGGATGTGCTGTCTGAA	GGCTCTTTCCTCTCCTTCAGG

### Gas Exchange and Chlorophyll Fluorescence Measurements

Gas exchange and fluorescence determination were carried out using the LI-6400-40 portable photosynthesis system equipped with an integrated fluorescence chamber head (Li-Cor, Lincoln, NE, United States) at the end of the treatment period with ANE (0 DAD) and at 1, 2, 3, and 4 days after the dehydration (1–4 DAD). Measurements were performed on fully expanded and exposed leaves from nine individual plants, three from each tray, for both treated and untreated plants, as previously described (Flexas et al., 2007). Instantaneous measurements of steady state photosynthetic CO_2_ assimilation rate (A), stomatal conductance (g_s_), intercellular CO_2_ concentration (C_i_) and transpiration rate (E) were determined at CO_2_ concentration of 400 μmol mol^-1^, relative humidity ranging between 45 and 55%, leaf temperature of 25°C and growing light intensity of 100 μmol m^-2^ s^-1^, as described in Fiorini et al. (2016). Determination of the actual photon yield of PSII photochemistry (Φ_PSII_) and the NPQ were carried out in the same experimental conditions at both growing (100 μmol m^-2^ s^-1^) and under excess light exposure (1000 μmol m^-2^ s^-1^). Intrinsic and instantaneous WUE were calculated as A to g_s_ ratio (A/g_s_) and A to E ratio (A/E), respectively. Leaves were allowed to adapt inside the chamber to the above conditions for about 5 min for adjustment and stabilization of the gas exchange and fluorescence parameters, as previously described (Scartazza et al., 2016). The values of Φ_PSII_ and NPQ were determined at steady state as in Moles et al. (2016). Briefly, Φ_PSII_ was calculated as Φ_PSII_ = (Fm′- F′)/Fm′, where Fm′ is the maximum fluorescence yield determined during exposition to actinic light after superimposing a saturating light flash and F′ is the fluorescence at the actual state of PSII reaction centers during actinic illumination. The NPQ was calculated according to the Stern–Volmer equation as NPQ = (Fm/Fm′)-1 (Bilger and Björkman, 1990). The maximum PSII photochemical efficiency (Fv/Fm) and the dark respiration were evaluated in plants adapted to dark for at least 30 min. The values of Fv/Fm were calculated as Fv/Fm = (Fm-Fo)/Fm′, where Fm and Fo are the minimal and the maximum fluorescence yield emitted by the leaves in the dark adapted state, respectively. The mesophyll conductance (g_m_) was calculated using the variable J method, as described by Harley et al. (1992) and Loreto et al. (1992). Briefly, this method is based on the simultaneous measurements of gas exchange and fluorescence parameters, comparing the electron transport rate determined simultaneously by gas exchange and fluorescence. The electron transport rate (Jf) was estimated as Jf = Φ_PSII_ × PPFD × α × β, where PPFD is the incident light intensity, α is the actual fraction of absorbed light and β is the distribution of light between the two photosystems (Maxwell and Johnson, 2000). For α and β we used the mean value 0.84 (Björkman and Demmig, 1987) and 0.5 (Loreto et al., 1992), respectively. The J method is sensible to variations of the CO_2_ compensation point between photosynthesis and photorespiration (Γ^∗^) and of the respiration in the light (Rl). The Γ^∗^ value is considered a remarkably conservative parameter (Harley et al., 1992) and was calculated by means of the specific factor for Rubisco relative to annual herbs (Galmés et al., 2005). Respiration of dark-adapted leaves (see above) was taken as a proxy for Rl (Centritto et al., 2009, 2011).

### Statistical Analysis

In all the experiments, for the comparison between untreated and treated plants the one-way ANOVA (*P* < 0.05) was applied. Data analysis was performed using GraphPad Prism (GraphPad Software, San Diego, CA, United States). The figures were prepared by using the Sigma Plot software for windows version 12.0 (Systal Software, Inc., San Jose, CA, United States).

## Results

### ANE Improves *Arabidopsis* Tolerance to Drought Stress

**FIGURE 1:**
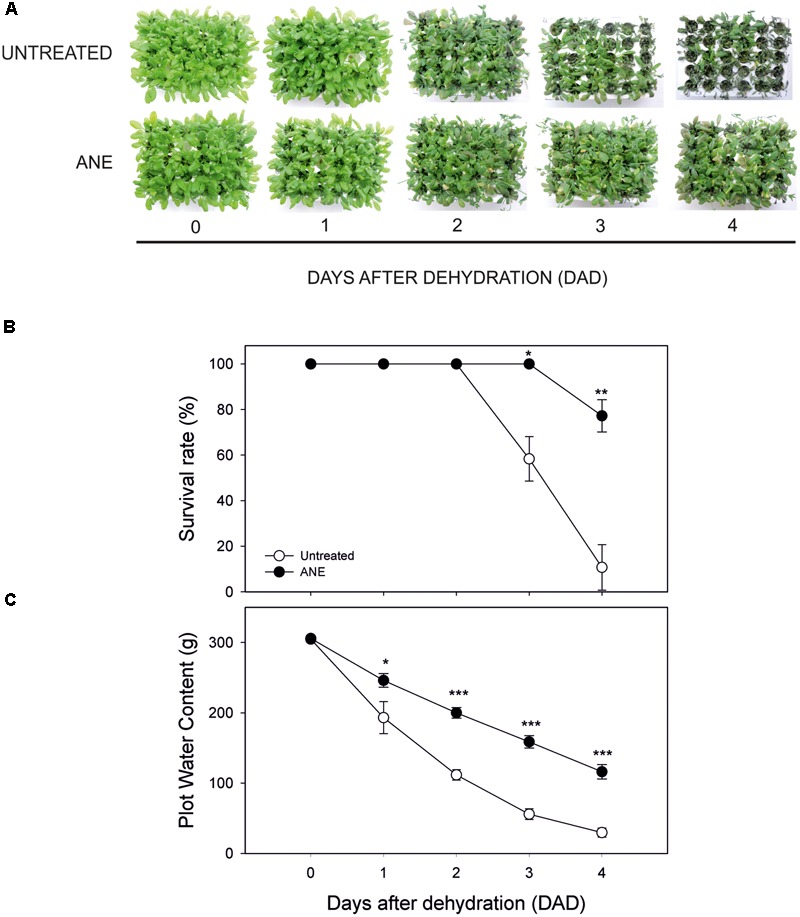
Phenotypical dehydration stress tolerance test, during 4 days of dehydration. Time 0 is the dehydration stress starting time and it corresponds exactly with the last day of *Ascophyllum nodosum* extract (ANE) treatment. Pictures of treated and not treated plants during 4 days after dehydration (0–4 DAD) **(A)**, plant survival rate **(B)** determined as mean ± SE of dead plants on three independent plots for both untreated and ANE-treated ones. Plot water content **(C)** was measured weighing three independent plots for both untreated and ANE-treated ones (see Materials and Methods). Untreated (white dot) and ANE-treated (black dot) samples were analyzed by one-way ANOVA (*P* < 0.05), ^∗^*P* ≤ 0.05; ^∗∗^*P* ≤ 0.01; ^∗∗∗^*P* ≤ 0.001.

We tested the effects of a pre-treatment with *Ascophyllum nodosum* extract (ANE) on *Arabidopsis* tolerance to drought stress (**Figure 1A**). The ANE was obtained by extraction of *Ascophyllum* alga and it contained various carbohydrates and amino acids as described in **Tables 2, 3**. The osmolarity of the nutrient solution remained unchanged after adding the ANE. Plants treated for 5 days with ANE did not show phenotypical differences compared to untreated ones (see untreated vs. ANE at time 0 in **Figure 1A**), but they were significantly different in terms of drought tolerance. At 3 DAD, untreated plants began to die while the ANE-treated ones were still alive (**Figure 1B**). Drought tolerance of ANE-treated plants was associated with a significantly higher water content of the plot/plants system throughout the treatment (**Figure 1C**). There were no significant differences in the RWC between untreated and ANE pre-treated plants up to 3 DAD. However, we were not able to record a decrease in the hydration status before the untreated plants began to die. At 4 DAD nearly 90% of the untreated plants were dead and the plants that were still alive showed a reduction in RWC. Conversely, ANE-treated plants, which were able to maintain their hydration level to 90% of RWC throughout the dehydration period, were only marginally affected in terms of survival rate (data not shown).

**Table 2:** Free monosaccharide and total monosaccharides quantified in ANE.

Analyte	% Free monosaccharides	% Total sugars
Mannose	0.0159 ± 0.0022	1.0939 ± 0.1232
Glucose	0.0511 ± 0.0041	2.1949 ± 0.2147
Galactose	0.0160 ± 0.0016	0.4264 ± 0.0114
Fucose	1.2272 ± 0.0254	8.0055 ± 0.2265
Xylose	0.4257 ± 0.0163	1.4978 ± 0.0384
Sum	1.7358 ± 0.03057	13.2185 ± 0.3379

**Table 3:** Free and total amino acids quantified in ANE.

Analyte	% Free amino acids	% Total amino acids
Ala	0,0112 ± 0.0036	0,1174 ± 0.0324
Gly	0,0083 ± 0.0021	0,1044 ± 0.0065
Ser	0,0017 ± 0.0009	0,0367 ± 0.0039
Pro	nd	nd
Val	nd	0.0171 ± 0.0033
Thr	0.0033 ± 0.0014	0.0429 ± 0.0091
Cys	nd	nd
Ileu	nd	0,0104 ± 0.0073
Leu	nd	0,0233 ± 0.0084
Asn	nd	nd
Asp	0.0697 ± 0.0018	0.2697 ± 0.0022
Gln	nd	nd
Glu	0.0267 ± 0.0011	0.8725 ± 0.1204
Met	nd	nd
His	nd	nd
Phe	0.0026 ± 0.0009	0.0254 ± 0.0047
Arg	0.0018 ± 0.0008	0.0264 ± 0.0036
Tyr	nd	0.0044 ± 0.0053
Trp	nd	nd
Lys	nd	0.02646 ± 0.0051

### Gas Exchanges and Gene Expression

**FIGURE 2:**
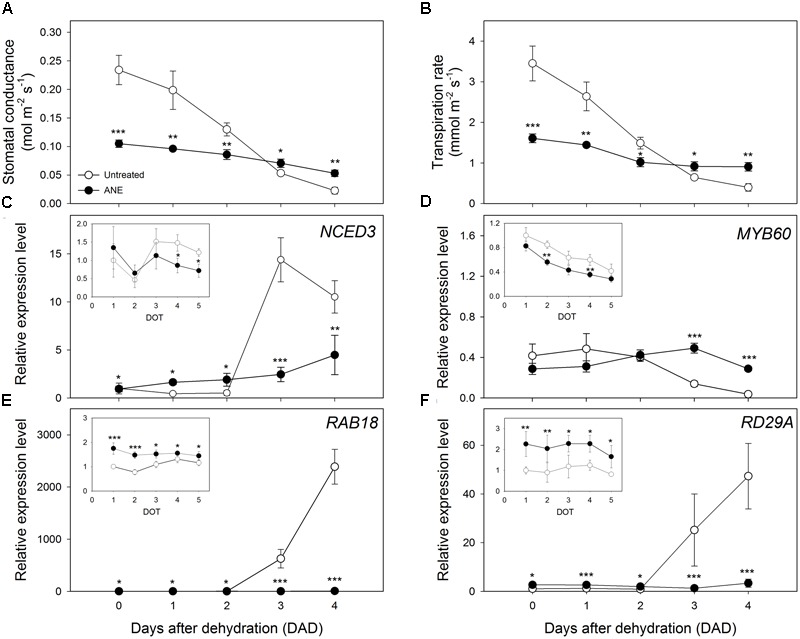
Modulation of stomatal closure and water efflux in association with ABA synthesis and responsive gene expression analysis, during 4 days of dehydration. Time 0 is the dehydration stress starting time and it corresponds exactly with the last day of *Ascophyllum nodosum* extract (ANE) treatment. Measurement of stomatal conductance **(A)** and transpiration rate **(B)**, during 4 days after dehydration (0–4 DAD). Each dot shows the mean ± SE of nine biological replicates. Analysis with qPCR of genes expression during 5 days of treatment (1–5 DOT, inset) and 4 days after dehydration (0–4 DAD) for *NCED3* **(C)** *MYB60* **(D)** *RAB18* **(E)** and *RD29A* **(F)**. Each dot shows the mean ± SE of four biological replicates. Untreated (white dot) and ANE treated (black dot) samples were analyzed by one-way ANOVA (*P* < 0.05), ^∗^*P* ≤ 0.05; ^∗∗^*P* ≤ 0.01; ^∗∗∗^*P* ≤ 0.001.

Plants treated for 5 days with ANE (time 0) showed a partial stomatal closure, leading to a highly significant reduction (55%) of the stomatal conductance (g_s_) (**Figure 2A**), associated with an equally strong decrease (53%) of the transpiration rate (E) (**Figure 2B**) compared to untreated plants. During the dehydration period, untreated plants tended to close stomata in order to reduce water losses by transpiration, reaching g_s_ and E values close to those of ANE-treated plants (**Figures 2A,B**). Between 3 and 4 DAD, untreated plants showed significantly lower values of g_s_ and E than ANE.

To investigate the role of ABA in stomata regulation, we analyzed the expression level of genes related to ABA synthesis (**Figure 2C**) and signaling (**Figures 2D–F**). Nine-*cis*-epoxycarotenoid dioxygenase 3 (*NCED3*), a gene involved in ABA biosynthetic pathway, was unaffected during the first 3 days of pre-treatment with ANE, and only after 4 and 5 days of treatment (DOT) a higher expression level was measured in untreated plants (**Figure 2C**, inset). This trend changed when the drought stress started. The expression level of *NCED3* remained slightly higher in ANE-treated plants during the first 2 days of dehydration, thereafter it raised in untreated plants during the last 2 days of dehydration (3–4 DAD). The expression pattern of Myb domain protein 60 (*MYB60*), a gene involved in stomata regulation, was similar in untreated and ANE pre-treated plants, although ANE decreased *MYB60* expression (**Figure 2D**, inset). During drought, however *MYB60* transcript was significantly more abundant in ANE-treated than in untreated plants (see DOT 2 and 4 in **Figure 2D**, inset), mirroring the stomatal conductance pattern shown in **Figure 2A**. The expression of two of the most common ABA-responsive genes, Responsive to ABA18 (*RAB18*) and Responsive to desiccation 29A (*RD29A*), was significantly higher in ANE-treated plants for all the 5 days of pre-treatment (**Figures 2E,F** inset). During the drought-stress treatment, *RAB18* and *RD29A* transcript abundance significantly increased in untreated plants, while it was largely unaffected in the plants that were pre-treated with ANE (**Figures 2E,F**).

**FIGURE 3:**
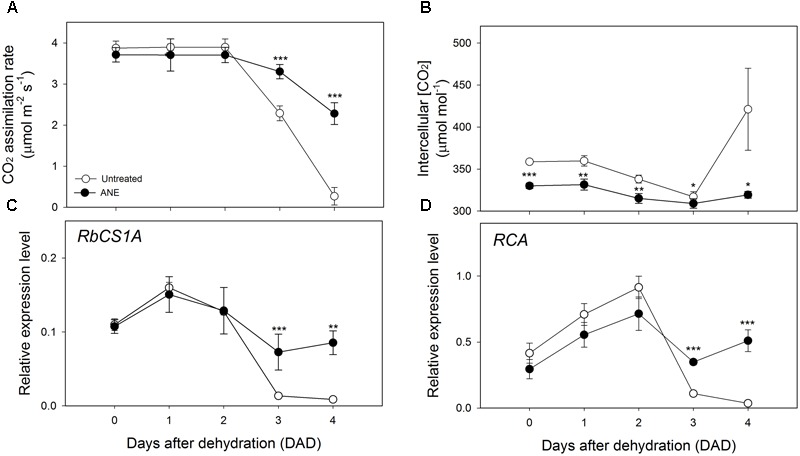
Photosynthetic CO_2_ uptake and modulation of intercellular CO_2_ concentration linked with the expression analysis of Rubisco quantity and activity genes. Time 0 is the dehydration stress starting time and it corresponds exactly with the last day of *Ascophyllum nodosum* extract (ANE) treatment. Measurements of CO_2_ assimilation rate **(A)** and intercellular CO_2_ concentration **(B)**, each dot shows the mean ± SE of nine biological replicates. Expression level analysis of *RBCS1A* **(C)** and *RCA* **(D)** during 4 days after dehydration (0–4 DAD). Each dot shows the mean ± SE of four biological replicates. Untreated (white dot) and ANE treated (black dot) samples were analyzed by one-way ANOVA (*P* < 0.05), ^∗^*P* ≤ 0.05; ^∗∗^*P* ≤ 0.01; ^∗∗∗^*P* ≤ 0.001.

The partial stomatal closure induced by the 5 days of treatment with ANE (**Figure 2A**, Time 0) caused only a slight and not significant reduction of CO_2_ assimilation rate (A) (**Figure 3A**) compared to untreated plants, while the intercellular CO_2_ concentration (C_i_) (**Figure 3B**) significantly decreased. The values of the CO_2_ assimilation rate remained almost unchanged during the first 2 days of dehydration, while, from 3 to 4 DAD, untreated plants showed a sharp decline of the photosynthetic CO_2_ uptake capacity, with significantly lower CO_2_ assimilation rate values, associated with higher C_i_. At 4 DAD, CO_2_ assimilation rate of untreated plants reached values close to 0 and C_i_ increased sharply.

**FIGURE 4:**
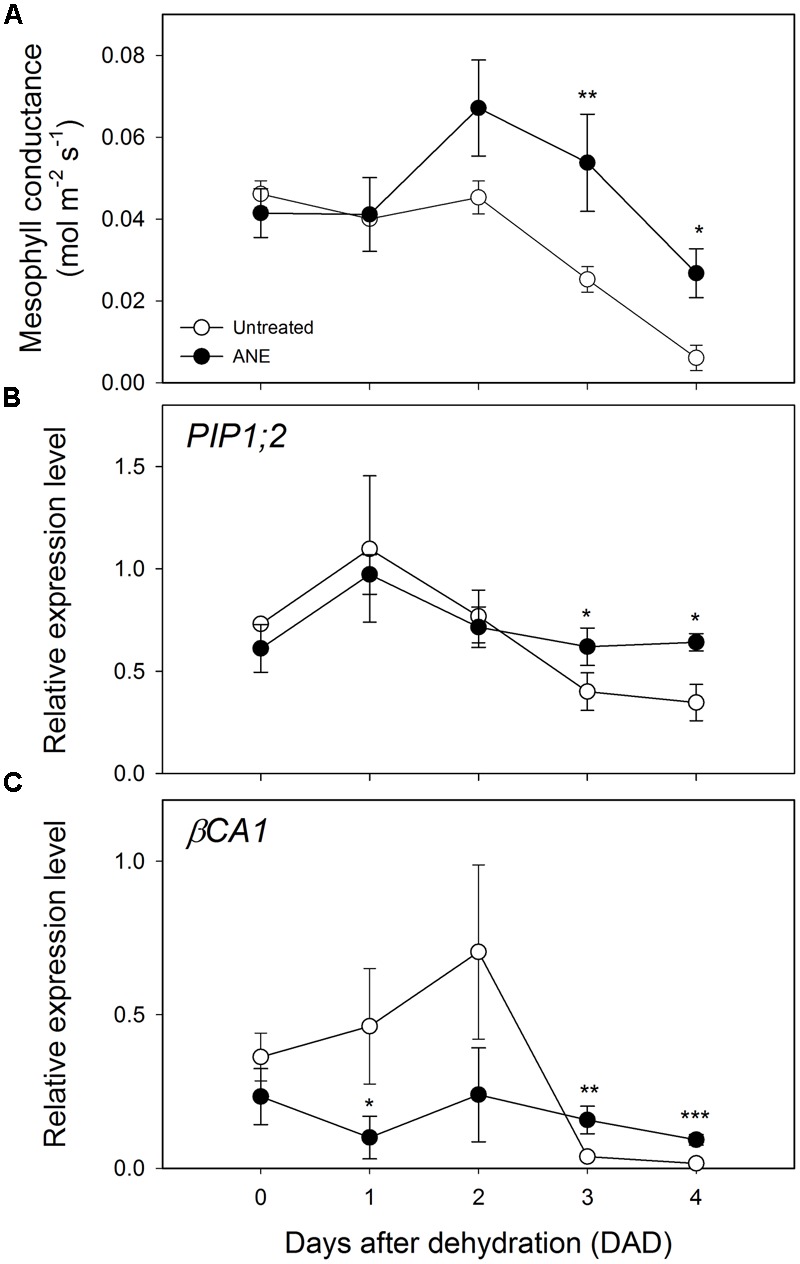
Modulation of mesophyll conductance and expression pattern of genes involved in CO_2_ diffusion from the sub-stomatal cavities to the carboxylation sites. Time 0 is the dehydration stress starting time and it corresponds exactly with the last day of *Ascophyllum nodosum* extract (ANE) treatment. Data of mesophyll conductance **(A)** during 4 days after dehydration (DAD). Each dot shows the mean ± SE of nine biological replicates. Data of expression level analysis of *PIP 1;2* **(B)** and *βCA1* **(C)**, during four DAD. Each dot shows the mean ± SE of four biological replicates. Untreated (white dot) and ANE treated (black dot) samples were analyzed by one-way ANOVA (*P* < 0.05), ^∗^*P* ≤ 0.05; ^∗∗^*P* ≤ 0.01; ^∗∗∗^*P* ≤ 0.001.

No significant differences in the expression level of the photosynthesis-related Ribulose 1,5-bisphosphate carboxylase/oxygenase small subunit A (*RBCS1A*) and Rubisco activase (*RCA*) was observed at the end of the ANE-pretreatment (**Figures 3C,D** Time 0). When plants were exposed to drought stress, a difference in the expression emerged after 3 DAD, with the ANE-treated plants showing a reduced attenuation of the expression of *RBCS1A* and *RCA* when compared to the untreated plants. The mesophyll conductance to CO_2_ (g_m_) was not significantly affected by the ANE treatment and did not show significant changes during the first 2 days of dehydration (**Figure 4A**). Conversely, between 3 and 4 DAD, g_m_ of untreated plants sharply decreased, maintaining significantly lower values than ANE-treated plants.

Two genes involved in the regulation of the mesophyll diffusional constrains are the aquaporin gene Plasma membrane Intrinsic Protein 1;2 (*PIP1;2*, **Figure 4B**) and β-Carbonic Anhydrase 1 (*βCA1*, **Figure 4C**), which can potentially alter the rate of final CO_2_ metabolized at the carboxylation sites. An up-regulation of *βCA1* was observed during the first 2 days of dehydration in untreated plants, followed by a strong decrease of expression level of both *PIP1;2* and *βCA1* at 3 and 4 DAD. Conversely, we noticed that ANE-treated plants showed an almost constant *PIP1;2* and *βCA1* level of expression throughout the dehydration period.

**FIGURE 5:**
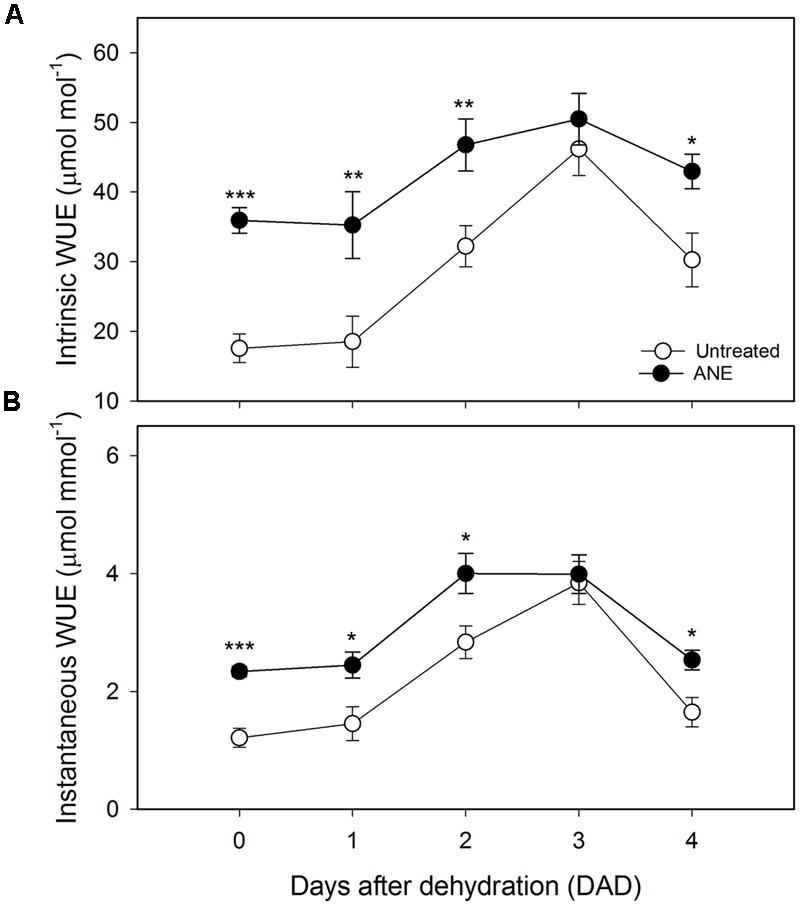
Water use efficiency (WUE) variation during the dehydration period. Time 0 is the dehydration stress starting time and it corresponds exactly with the last day of *Ascophyllum nodosum* extract (ANE) treatment. Measurement of intrinsic **(A)** and instantaneous **(B)** WUE for untreated and ANE-treated plants. Each dot shows the mean ± SE of nine biological replicates. Untreated (white dot) and ANE-treated (black dot) samples were analyzed by one-way ANOVA (*P* < 0.05), ^∗^*P* ≤ 0.05; ^∗∗^*P* ≤ 0.01; ^∗∗∗^*P* ≤ 0.001.

The treatment-induced reduction of g_s_ and E, associated with only a slight decrease of A, was reflected in a relevant increase in both intrinsic (+105%, **Figure 5A**) and instantaneous (+93%, **Figure 5B**) WUE with respect to untreated plants. From 1 to 3 DAD both untreated and ANE-treated plants tended to close stomata in order to reduce transpiration, leading to a strong increase in intrinsic and instantaneous WUE. However, ANE was able to maintain a higher WUE throughout the dehydration period, with the exception of 3 DAD when untreated and ANE-treated showed similar values.

**FIGURE 6:**
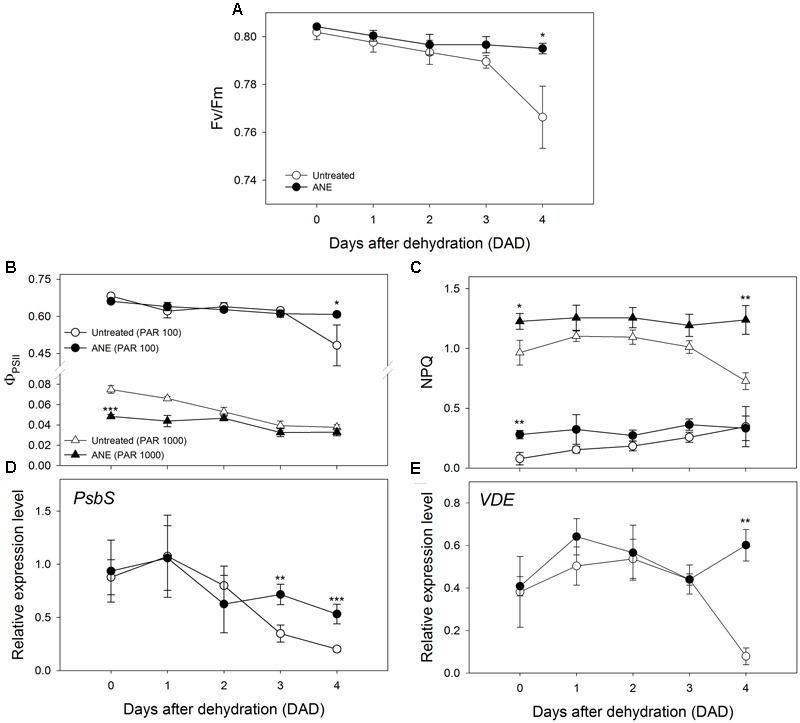
Chlorophyll a fluorescence measurements and expression levels of genes related to photoprotection mechanisms at photosystem II. Time 0 is the dehydration stress starting time and it corresponds exactly with the last day of *Ascophyllum nodosum* extract (ANE) treatment. Potential efficiency of PSII photochemistry (Fv/Fm, **A**), actual photon yield of PSII photochemistry in the light (ΦPSII, **B**), non-photochemical quenching (NPQ, **C**). ΦPSII and NPQ were determined at photosynthetically active radiation (PAR) of 100 and 1000 μmol m^-2^ s^-1^. Each dot shows the mean ± SE of nine biological replicates. Expression pattern of *PsbS* **(D)** and *VDE* **(E)** during 5 days of dehydration (0–4 DAD). Each dot shows the mean ± SE of four biological replicates. Untreated (white dot) and ANE-treated (black dot) samples were analyzed by one-way ANOVA (*P* < 0.05), ^∗^*P* ≤ 0.05; ^∗∗^*P* ≤ 0.01; ^∗∗∗^*P* ≤ 0.001.

Plants treated with ANE were able to maintain the potential efficiency of PSII photochemistry (Fv/Fm) close to the optimal value of 0.8 throughout the dehydration period; conversely, at 4 DAD, untreated plants exhibited a value of Fv/Fm below 0.8, significantly lower than ANE-treated plants (**Figure 6A**). In addition, immediately before the dehydration, ANE-treated plants showed a significantly lower actual photon yield of PSII photochemistry in the light (Φ_PSII_) than untreated plants, associated with a higher NPQ, at both growth (100 μmol m^-2^ s^-1^) and excess (1000 μmol m^-2^ s^-1^) light conditions (**Figures 6B,C**). From 0 to 4 DAD, Φ_PSII_ in the growth-chamber light condition decreased, of about 29 and 8% in untreated and ANE-treated plants respectively, with a corresponding NPQ increase of about 78 and 16%. Under excess light exposure, both untreated and ANE-treated plants showed a strong reduction of Φ_PSII_ associated with an increase of NPQ compared to growth light conditions. Moreover, Φ_PSII_ at high light irradiance was reduced during the dehydration period, showing a decrease of about 50 and 32% from 0 to 4 DAD in untreated and ANE-treated plants, respectively. However, at 4 DAD, untreated plants were unable to safely dissipate the excessive excitation energy at PSII when subjected to high irradiance levels, as suggested by the decrease of both NPQ and Fv/Fm compared to ANE.

The expression pattern of two genes involved in photo-protective mechanisms, Photosystem II subunit S (*PsbS*, **Figure 6D**) and Violaxanthin de-epoxidase (*VDE*, **Figure 6E**) did not show significant differences during the pre-treatment period (data not shown). During dehydration significant differences occurred in the last part of the stress period, when the amount of transcripts was higher in ANE than in untreated plants for *PsbS* (3–4 DAD) and for *VDE* (4 DAD).

**FIGURE 7:**
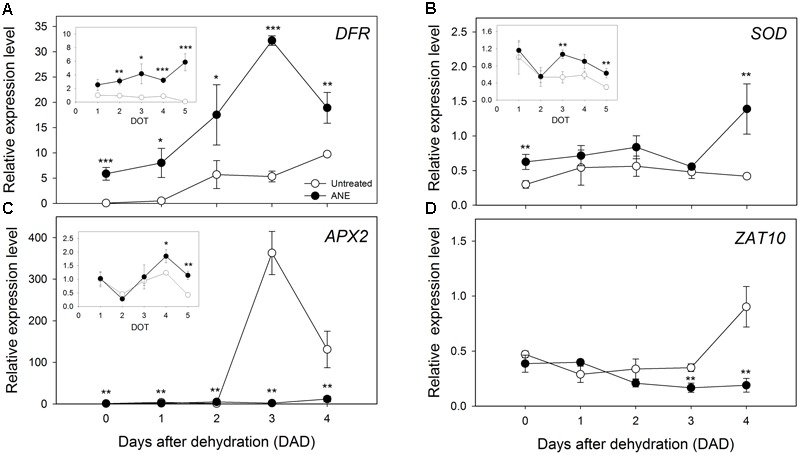
Pattern of expression level for genes involved in ROS scavenging and responsive processes. Time 0 is the dehydration stress starting time and it corresponds exactly with the last day of *Ascophyllum nodosum* extract (ANE) treatment. Expression analysis of *DFR* **(A)**, *SOD* **(B),** and *APX2* **(C)** for 5 days of treatment (1–5 DOT, inset) and from 0 to 4 days after dehydration (0–4 DAD). Expression analysis of *ZAT10* **(D)** from 0 to 4 DAD. Each dot shows the mean ± SE of four biological replicates. Untreated (white dot) and ANE-treated (black dot) samples were analyzed by one-way ANOVA (*P* < 0.05), ^∗^*P* ≤ 0.05; ^∗∗^*P* ≤ 0.01; ^∗∗∗^*P* ≤ 0.001.

Drought stress often induces the synthesis of anthocyanins, playing a positive role in *Arabidopsis* tolerance to drought (Nakabayashi et al., 2014). The expression of Dihydroflavonol reductase (*DFR*), a gene involved in the late biosynthetic step of anthocyanins, was higher in ANE-treated than in untreated plants, already at the end of the ANE-pre-treatment as well as during the dehydration period (**Figure 7A**). A similar trend was observed also for the expression level of a gene involved in ROS scavenging processes, namely Superoxide dismutase (*SOD*, **Figure 7B**). The expression of Ascorbate Peroxidase 2 (*APX2*) was instead higher in ANE pre-treated plants (**Figure 7C** inset) but only untreated plants showed a sharp increase in *APX2* expression (**Figure 7C**). The high expression of *APX2* suggested the occurrence of oxidative stress in untreated plants, an instance confirmed by the pattern of expression of *ZAT10* (**Figure 7D**).

## Discussion

Acclimation to drought involves metabolic and morphological alterations that help plants to prevent permanent damages. These mechanisms can be induced in plant by treatment with seaweed-based products, increasing the overall tolerance to abiotic stresses (Spann and Little, 2011; Petrozza et al., 2014; Xu and Leskovar, 2015). Our results showed higher tolerance to drought stress in plants that were pre-treated with ANE. The ANE treatment may have imposed a lenient stress on the plants, allowing them to be better prepared for the following severe stress treatment. Higher tolerance was the likely consequence of stomata closure in ANE-treated plants, preventing excessive water losses. Closure of stomata represents the primary plant defense against drought stress, in order to save water and maintain turgor (Chaves and Oliveira, 2004; Skirycz and Inzé, 2010). Indeed the RWC was kept high in all plants, indicating that stomata closure is effective in maintaining an adequate water content even in drought stressed plants. Also the untreated plants tend to close stomata during the dehydration period in order to reduce water losses and maintain a high hydration level, although this mechanism was not sufficient to prevent their death. In fact, we recorded a drop of survival rate before we observed a decrease in RWC, possibly due to the severity and speed of the imposed stress.

The regulation of stomatal movement is strictly dependent on several environmental conditions and responds to a wide range of stimuli, from intracellular signaling to environmental factors (Hetherington and Woodward, 2003). *MYB60* is directly involved in the regulation of stomatal movements, it is down regulated by ABA, and its induction presumably facilitates the stomata opening (Cominelli et al., 2005). The reduced g_s_ in ANE-treated plants highlighted that the treatment stimulated a partial stomatal closure, through lower expression of *MYB60*, training plants to the incoming stress. The expression of *NCED3, RAB18* and *RD29A* increased sharply during the last 2 days of dehydration, when a steep decrease of stomatal conductance was recorded. These results suggested a strong synthesis of ABA in order to close stomata and counteract the water loss. The late expression of *NCED3* in untreated plants is suggestive of ABA biosynthesis, playing a late role in drought stress response in untreated plants. The expression of *RAB18* and *RD29A*, both highly ABA-responsive, supports the assumption that *de novo* ABA synthesis is not a major player in the decreased g_s_ observed during the dehydration treatment. In addition, the lower expression of these genes, identified as specific drought marker genes, in the ANE pre-treated plants highlighted as the treatment induced a lower water stress perception. It is well-known that several signaling, other than ABA, can modulate stomatal conductance. These include other hormones (jasmonate, salicylic acid, ethylene), polyamines and microbial elicitors, inducing production of nitric oxide (NO) and ROS in guard cells (Zhang et al., 2001; Allègre et al., 2009; Srivastava et al., 2009; Gayatri et al., 2013; Ye and Murata, 2016). Hence, it is possible that signals other than ABA, or ABA like molecules (Spann and Little, 2010), might be induced by the treatment provoking the observed partial stomatal closure. For this reason, the potential priming role of salicylic acid, jasmonate, ROS and NO signaling pathways were investigated, by analyzing the expression analysis of genes involved in these pathways. However, the expression pattern for *PR1* (SA), *PDF1*.*2* (JA), *ZAT10* (ROS), *NOA1* (NO) were not induced by the ANE pre-treatment (data not shown). It is thus tempting to speculate that the most reliable cause of the observed responses in ANE-treated plants is a higher sensitivity of stomata to relatively low changes in ABA concentrations, as previously proposed (Trejo and Davies, 1991; Du et al., 2012).

The partial stomatal closure triggered by the treatment induced a strong decrease of the transpiration rate, associated with only a slight reduction of net photosynthesis. This could be explained taking into account that plants were growing at low light intensity (100 μmol m^-2^ s^-1^). At this low irradiance level, photosynthesis is mostly limited by the absorbed light and not by the CO_2_ concentration at the carboxylation site, in absence of any damages at the photosynthetic apparatus. On the other hand, our data indicated that the treatment did not impair the CO_2_ transfer within the mesophyll and the carboxylation efficiency (see below), as suggested also by the low intercellular CO_2_ concentration. Consequently, the treatment with ANE leaded to a relevant increase of both intrinsic and instantaneous WUE. At higher light condition (1000 μmol m^-2^ s^-1^), a decrease of g_s_ and an increase of WUE at T0 was still observed in ANE-treated plants, although at this light exposure the photosynthetic rate was reduced compared to untreated plants; however, pre-treatment with ANE still induced a higher tolerance of photosynthetic apparatus to drought stress (Santaniello and Scartazza, unpublished). Improving WUE is one of the major goal for the future research to ensure higher crop yields against unfavorable environmental stresses (Flexas et al., 2016). Hence, the use of products in agriculture able to trigger priming signals, which modulate stomatal conductance and regulate water loss, can improve WUE and the drought tolerance in crops (Du et al., 2012). In response to dehydration, all the plants tended to close their stomata and increase WUE, but for untreated, on the contrary to ANE-treated plants, this mechanism was not sufficient to prevent damages to the photosynthetic apparatus. In fact, our results highlighted non-stomatal detrimental drought effects on photosynthetic machinery of untreated plants after 3 days of dehydration stress. At this time point, a decrease in the expression levels of both *RBCS1A* and *RCA* was observed. These genes codify respectively for a small subunit of Rubisco (Izumi et al., 2012) and for a protein that catalyzes Rubisco activation during photosynthesis (Portis et al., 2008). The decline in the total mRNA level for both these genes suggested a possible reduced carboxylation capacity in untreated with respect to ANE-treated plants during the last phases of dehydration, supporting the hypothesis that damages to photosynthetic apparatus induced by dehydration were alleviated by pre-treatment with ANE. In addition to stomatal modulation and biochemical limitations, also the diffusion of CO_2_ from sub-stomatal cavities to the site of carboxylation is crucial for the regulation of photosynthesis under water stress (Grassi and Magnani, 2005; Keenan et al., 2010; Flexas et al., 2012). In our experiments, in agreement with the reduction in carboxylation capacity, untreated plants showed a drought-induced reduction in the mesophyll conductance to CO_2_. This caused an increase in the diffusional limitation of CO_2_ within the mesophyll that, finally, reduced its availability within the chloroplasts for Rubisco.

The reduction in g_m_ could be related to the decreased expression level of the genes *PIP1;2* and *βCA1*, which are considered the most likely candidate genes for the regulation of CO_2_ diffusion within the mesophyll (Flexas et al., 2006; Heckwolf et al., 2011; Kawase et al., 2013). An up-regulation of *βCA1* was observed during the first 2 days of dehydration in untreated plants. This result suggests that untreated plants tend to modulate the expression level of *βCA1* during the first phase of dehydration in order to counteract the reduced photosynthetic rate and the consequent oxidative damages (Das et al., 2016). Subsequently, the expression level of both *βCA1* and *PIP1;2* drastically decreased during the last phases of dehydration, associated with the drop in CO_2_ assimilation rate and g_m_. Conversely, plants pre-treated with ANE did not substantially modify the expression level of both *βCA1* and *PIP1;2* throughout the dehydration period and were able to maintain almost unaltered g_m_. Hence, the higher expression of *βCA1* and *PIP1;2*, recorded in ANE-treated plants during the last phases of dehydration, could contribute in maintaining a quite constant supply of CO_2_ for Rubisco and a lower intercellular CO_2_ concentration. Both biochemical and diffusional constrains strongly reduced the photosynthetic capacity in drought-stressed untreated plants, which at 4 days after dehydration showed an assimilation rate close to 0, an increase of the sub-stomatal CO_2_ concentration and a decrease of the potential photochemical efficiency. This confirms possible damages to the photosynthetic apparatus. Conversely, ANE pre-treated plants were able to maintain a strong stomatal control (Fan et al., 2011) and relatively higher values of both WUE and g_m_ during the last 2 days of dehydration in comparison with untreated plants. Consequently, they exhibited a significantly higher photosynthetic capacity, without showing relevant signs of photo-damage and photo-inhibition or significant reduction in the expression levels of photosynthetic-related genes. In fact, to prevent permanent damages to photosynthetic apparatus, caused by the excess of energy during drought stress, highly efficient energy dissipative processes are necessary (Sonoike, 2011; Vass, 2012). In this respect, ANE-treated plants showed a higher ability to dissipate the excess of energy as heat in the reaction centers of PSII, as suggested by the higher NPQ values. This ability is even highly evident under exposure to high irradiance level, when plants need to safely dissipate a greater proportion of excessive energy (Demmig-Adams and Adams, 2000). Instead, the NPQ of untreated plants tended to increase in the first stress days, but at the end of the stressed time, it decreased under excessive light condition notwithstanding the reduction in the photochemical activity (Moles et al., 2016). It is well-known that the protein PsbS and the enzyme Violaxanthine de-epoxidase (VDE) are involved in the activation of non-radiative energy dissipation mechanisms, inducing a change in the conformation of photosynthetic membranes that triggers thermal energy dissipation (Demmig-Adams and Adams, 2000; Li et al., 2000). The sharp decrease in the expression pattern of both these genes supported the hypothesis that in untreated plants, differently from ANE-treated ones, the dehydration impaired the energy dissipation mechanisms provoking photo-inhibition and irreversible photo-damages at the photosynthetic apparatus. Under high radiative pressure, the excess of energy at PSII is not readily processed by photochemistry, hence it can induce the production of triplet excited states of chlorophyll. In order to counteract the resulting production of ROS, an efficient antioxidant system is necessary to protect the photosynthetic apparatus (Krieger-Liszkay, 2005). Our data indicated that ANE pre-treatment induced the activation of the antioxidant defense system (Fan et al., 2011) that, in combination with an efficient energy dissipation mechanism, was able to avoid irreversible damages to PSII. This hypothesis is supported by the expression trend of genes involved in ROS scavenging processes such as *DFR* and *SOD*, which indicated a higher capacity of ANE-treated plants to deal with oxidative stress. Interestingly, these genes were more-expressed in ANE-treated plants already before the dehydration, possibly inducing a priming effect to counteract the incoming stress and, hence, alleviating drought-induced oxidative damages. Overall, our data indicate that pre-treatment with ANE could represents a potential tool for farmers to alleviate the damages of short-term periods of severe drought stress by inducing an improvement of WUE and stimulating the antioxidative and photoprotective defense systems.

## Conclusion

Dehydration strongly affected the photosynthetic performance of untreated plants acting on both stomatal and non-stomatal constrains, while *Arabidopsis* plants treated with ANE were able to maintain a relatively higher photochemical and non-photochemical energy dissipation capacity for longer time during dehydration, thus preserving the photosynthetic apparatus from irreversible photo-damages. The extract chemical composition of ANE in unknown, but it is very likely that several bioactive molecules are extracted together with carbohydrates and aminoacids. Some of these molecules are possibly activating signaling pathways leading to stomata closure and, as a consequence enhanced tolerance to water stress. We cannot exclude that this mechanism serves to acclimatize the plants against subsequent drought stress. The effect of these molecules present in ANE are likely also to affect photosynthetic efficiency, given that ANE plants showed improved photosynthetic efficiency at low stomata conductance of the leaves. Overall, our results suggest that pre-treatment with ANE is effective in promoting an increase in WUE and dehydration tolerance, through priming stomatal modulation and antioxidative defense systems, thus conferring to the primed plants the capability to be more prone to counteract short-term periods of severe water stress.

Our results point out the need to better understand the effects of ANE treatment on stimulation of plant defense responses to water stress, considering the complex interactions among irradiance level, phenology and severity of stress. Biochemical analyses of metabolite compounds are necessary to unravel the mechanisms controlling the ANE-mediated effects to drought. Finally, it would be relevant to evaluate the role of all the chemical components present in the ANE extract on the observed responses. This would be of particular relevance for the development of new prototypes aimed to alleviate drought stress in crops.

## Author Contributions

The work was conceived and designed by PP, EL, ASa, and ASc. AB, DDT, and AP produced and provided the *Ascophyllum nodosum* extract (ANE). EL, FG, and ASa setup the hydroponic system for growing plants and performed treatments with ANE, collecting the phenotypical and gene expression analysis data. ASc performed gas exchanges measurements and further analysis of data. ASa and ASc drafted the manuscript, which was critically revised by all authors.

## Conflict of Interest Statement

The authors declare that the research was funded by Valagro SpA, a company involved in the production of ANE. The funder was not involved in the study design or collection, analysis or interpretation of the data.

## Footnotes

1 www.ambion.com

2 www.bio-rad.com

3 https://genorm.cmgg.be

4 http://quantprime.mpimp-golm.mpg.de
